# *Micrurus *snake venoms activate human complement system and generate anaphylatoxins

**DOI:** 10.1186/1471-2172-13-4

**Published:** 2012-01-16

**Authors:** Gabriela D Tanaka, Giselle Pidde-Queiroz, Maria de Fátima D Furtado, Carmen van den Berg, Denise V Tambourgi

**Affiliations:** 1 Immunochemistry Laboratory, Butantan Institute, Av. Vital Brazil, 1500, São Paulo, 05503-900, Brazil; 2 Herpetology Laboratory, Butantan Institute, Av. Vital Brazil, 1500, São Paulo, 05503-900, Brazil; 3 Department of Pharmacology, Oncology and Radiology, School of Medicine, Cardiff University, Cardiff CF14 4XN, UK

## Abstract

**Background:**

The genus *Micrurus*, coral snakes (Serpentes, Elapidae), comprises more than 120 species and subspecies distributed from the south United States to the south of South America. *Micrurus *snake bites can cause death by muscle paralysis and further respiratory arrest within a few hours after envenomation. Clinical observations show mainly neurotoxic symptoms, although other biological activities have also been experimentally observed, including cardiotoxicity, hemolysis, edema and myotoxicity.

**Results:**

In the present study we have investigated the action of venoms from seven species of snakes from the genus *Micrurus *on the complement system in *in vitro *studies. Several of the *Micrurus *species could consume the classical and/or the lectin pathways, but not the alternative pathway, and C3a, C4a and C5a were generated in sera treated with the venoms as result of this complement activation. *Micrurus *venoms were also able to directly cleave the α chain of the component C3, but not of the C4, which was inhibited by 1,10 Phenanthroline, suggesting the presence of a C3α chain specific metalloprotease in *Micrurus *spp venoms. Furthermore, complement activation was in part associated with the cleavage of C1-Inhibitor by protease(s) present in the venoms, which disrupts complement activation control.

**Conclusion:**

*Micrurus *venoms can activate the complement system, generating a significant amount of anaphylatoxins, which may assist due to their vasodilatory effects, to enhance the spreading of other venom components during the envenomation process.

## Background

The Elapidae family is represented in America by three genera of coral snakes: *Micruroides, Leptomicrurus *and *Micrurus*, the latter being the most abundant and diverse group. In Brazil, *M. corallinus *and *M. frontalis *are responsible for the majority of coral snake envenomations. Although, *Micrurus *bites are relatively rare, the accidents can cause death, by muscle paralysis and respiratory arrest, few hours after envenomation [1].

The main feature of the coral snake action is the neurotoxicity, although, experimentally, it has been documented that some *Micrurus *venoms may produce myotoxicity and local lesions [2,3]. Furthermore, many enzymatic activities were detected in *Micrurus *venoms including phospholipase A_2_, hyaluronidase, phosphodiesterase, leucine amino peptidase, L-amino acid dehydrogenase and L-amino acid oxidase activities [4-6]. Only little or no proteolytic effects have been detected in *Micrurus *venoms [5,6].

Previously, we have analyzed the pro-inflammatory properties of the snake venoms from the Elapidae family, including the genera *Micrurus *(*M. ibiboboca *and *M. spixii*) and *Naja *(*N. naja, N. melanoleuca *and *N. nigricollis*), and demonstrated that these snake venoms can activate the complement system in normal serum [7]. The electrophoretic conversion of C3 was observed with all venoms in human normal serum containing normal concentrations of Ca^2+ ^and Mg^2+^. However when Ca^2+ ^was chelated the conversion of C3 was only observed in serum incubated with the *N. naja *and *N. melanoleuca *venoms. Purified human C3 was electrophoretically converted, in the absence of other complement system components, by the venoms from *N. naja, N. nigricollis *and *M. ibiboboca*. However, only the venoms from *N. naja *and *N. melanoleuca *contained a 144 kDa protein recognized by anti-sera against cobra venom factor or human C3.

The complement system (C) consists of three activation pathways, *i.e*., classical (CP), alternative (AP) and lectin (LP) pathways, which converge at the proteolytic activation step of C3, the central component of the complement system. Complement activation may generate anaphylatoxins and membrane attack complex (MAC). Anaphylatoxins (C3a, C4a, and C5a) are considered the bridge between the innate and adaptive immunity, and they are responsible for control the local pro-inflammatory response through vasodilatation and the chemotaxis and activation of leukocytes [8-11]. The regulatory mechanisms of complement are extremely balanced, controlled by several complement inhibitors, such as membrane-bound complement regulators, and plasma proteins like C1-esterase inhibitor (C1-INH), which regulates activation of C1 and MASPs of the classical and lectin complement pathways, among others [8,12,13].

The complement system not only plays an important role in the defense system, but also contributes to the amplification of inflammation if activated in excess or inappropriately controlled. The best-characterized venom that has a complement activating substance is that of the Cobra (*Naja naja*, Elapidae). Cobra venom contains a C3-like molecule named Cobra venom factor (CVF), which can initiate the alternative pathway by forming with factor B an extremely stable C3/C5 convertase and the consequence of this is excess C5a generation and C-consumption [14-16]. We have recently investigated the complement activating properties of snake venoms of the *Bothrops *genus and found that several venoms caused complement activation [17]. The aim of the present study was to further investigate the action of snake venoms from the genus *Micrurus*, occurring in Brazil, on the activation pathways and components of the complement system.

## Results

### Action of *Micrurus *spp venoms on the complement system activation pathways

**Figure 1:**
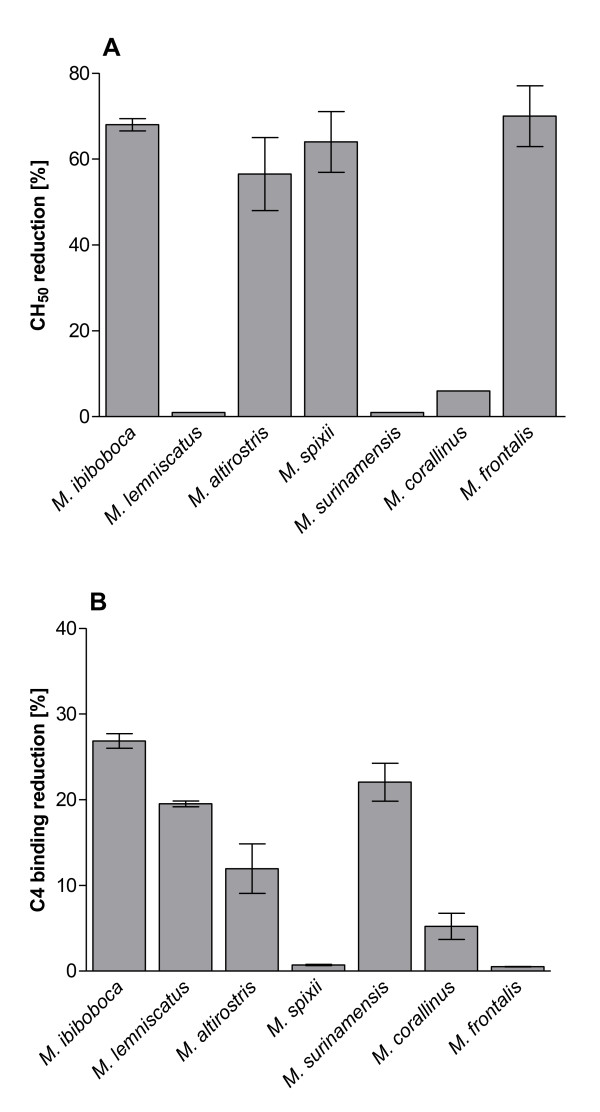
**Action of *Micrurus *spp venoms on the complement pathways**. Samples (50 μl) of normal human serum (NHS), as complement source, were incubated with 50 μl of *Micrurus *spp venoms (50 μg) or PBS for 30 min at 37°C. The residual complement lytic activity of the mixtures was measured using antibody-sensitized sheep erythrocytes, as targets, for classical pathway **[A]**. After incubation for 1 h at 37°C, the absorbance of the supernatants was measured at λ414 nm and the percentage of haemolysis calculated. The residual lectin pathway complement activity **[B] **was determined on ELISA plates coated with mannan. Data are representative for three separate experiments and expressed as mean of duplicates +/-SD. Results were expressed as reduction of percentage of haemolysis [for A] or reduction of C4 binding [B] in relation to the control samples (NHS + buffer).

In order to evaluate the possible action of *Micrurus *spp venom toxins on the complement system, samples of normal human serum were incubated with PBS or the venoms and the remaining complement lytic activity was measured under conditions to develop the classical, alternative or lectin pathways. Figure 1A shows that the venoms from *M. ibibococa, M. altirostris, M spixii *and *M. frontalis *induced a significant reduction of the classical complement pathway lytic activity, as measured in haemolytic assays using antibody-sensitized sheep erythrocytes. Analysis of the activity of *Micrurus *spp venoms on the lectin pathway showed that *M. ibiboboca, M. lemniscatus, M. altirostris, M. surinamensis *and *M. corallinus *snake venoms induced a low but significant reduction of the LP activity (Figure 1B). No action was observed of *Micrurus *spp venoms on the alternative pathway (data not shown).

**Figure 2:**
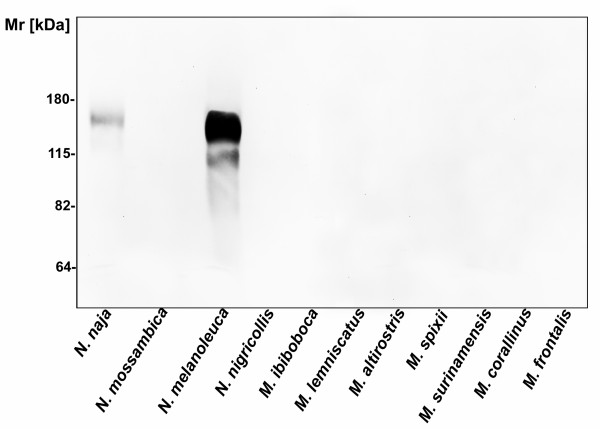
**Analysis of the presence of CVF components in Elapidae snake venoms**. Samples of 10 μg of the *Micrurus *spp and *Naja *spp venoms were solubilised in non-reducing sample buffer and run on 10% SDS-PAGE gel. The presence of CVF components was evaluated by Western blot using anti-CVF rabbit antiserum, diluted 1:1500.

To investigate if the decrease in the pathways activities, induced by *Micrurus *venoms, could be due to the presence of Cobra Venom Factor (CVF) like components, venoms were analyzed by western blotting using an antiserum raised against CVF from *N. naja*. Figure 2 shows that CVF components were clearly detected in *N. naja *and *N. melanoleuca *venoms. On the other hand, no CVF antigenically related components were detected in *N. nigricollis, N. mossambica *or *Micrurus *spp venoms.

### Induction of the anaphylatoxins by *Micrurus *spp venoms

**Figure 3:**
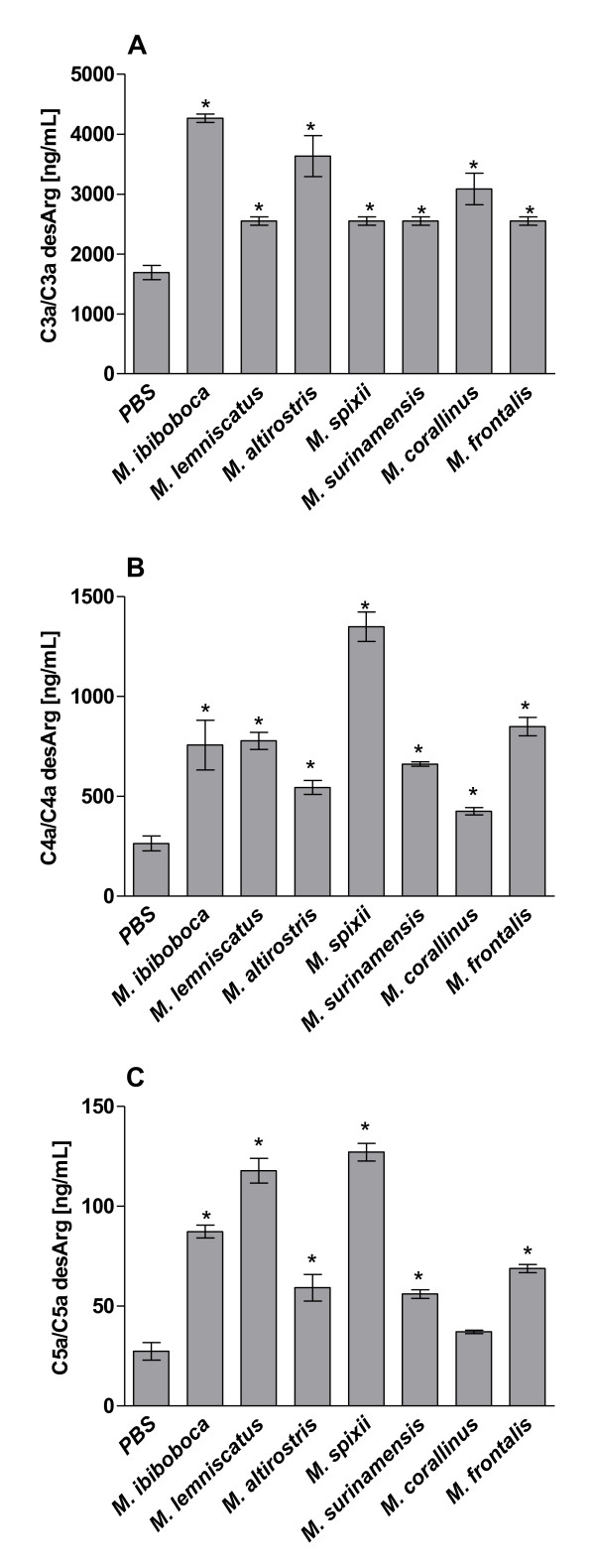
**Generation of human serum anaphylatoxins induced by *Micrurus *spp venoms**. Samples (50 μl) of normal human serum (NHS), as complement source, were incubated with 50 μl of *Micrurus *spp venoms (50 μg) or PBS for 30 min at 37°C. The generation of the anaphylatoxins (C3a, C4a and C5a) in the serum samples was measured by Cytometric Bead Array. Data are representative for three separate experiments and expressed as mean of duplicates +/-SD; results were expressed as concentration of each anaphylatoxin per mL of human serum.

To assess if the above observed reduction in complement activities was caused by C-inhibition or C-activation/consumption, the generation of C-activation products C3a, C4a and C5a was measured. Figure 3 shows that the majority of *Micrurus *spp venoms induced the production of significant amounts of the three anaphylatoxins. *M. corallinus *venom did not induce C5a release, but considerable amounts of C3a and C4a. These data suggest that the reduction in CP and LP, as observed above, is due to C-activation/consumption rather that C-inhibition.

### *Micrurus *spp venoms cleave the C3α chain

**Figure 4:**
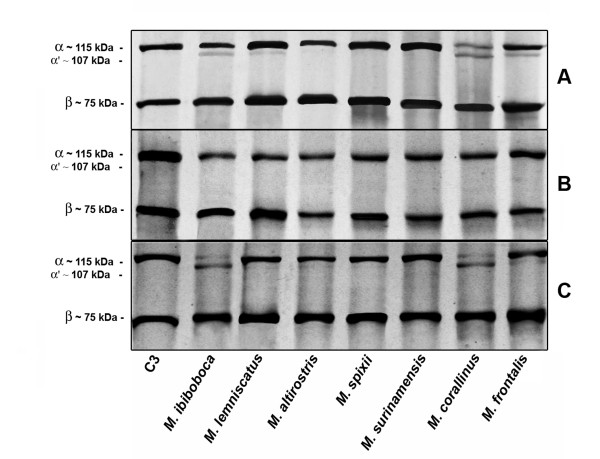
**Proteolytic action of the *Micrurus *spp venoms on human purified C3**. **[A] **Samples of purified component C3 (1 μg) were incubated with 2 μg of *Micrurus *spp venoms or PBS for 30 min at 37°C. Inhibition assays were performed by including 10 mM of 1,10 phenanthroline **[B] **or PMSF **[C] **in the mixtures. Cleavage was visualized by western blot using anti-C3 serum.

In order to analyze the possibility of direct proteolytic action of *Micrurus *venoms on the complement molecules, purified samples of C3 were incubated with the venoms and analyzed by western blot for the presence of cleavage fragments. Figure 4A shows that the venoms from *M. ibiboboca, M. lemniscatus, M. corallinus, M. frontalis *were able to induce cleavage of the C3 α chain (M_r _~115 kDa), but not of the β chain, generating a fragment with an estimated M_r _of 107 kDa. The C3 α chain cleavage, induced by the venoms, could be prevented by the use of 1,10 phenanthroline, a metalloproteinase inhibitor (Figure 4B), but not by PMSF, a serineprotease inhibitor (Figure 4C). Analysis of the *Micrurus *venoms action on purified human C4 showed that all venoms were unable to induce cleavage of the α, β or γ chains (data not shown).

### *Micrurus *spp venoms induce cleavage of the C1-Inhibitor

**Figure 5:**
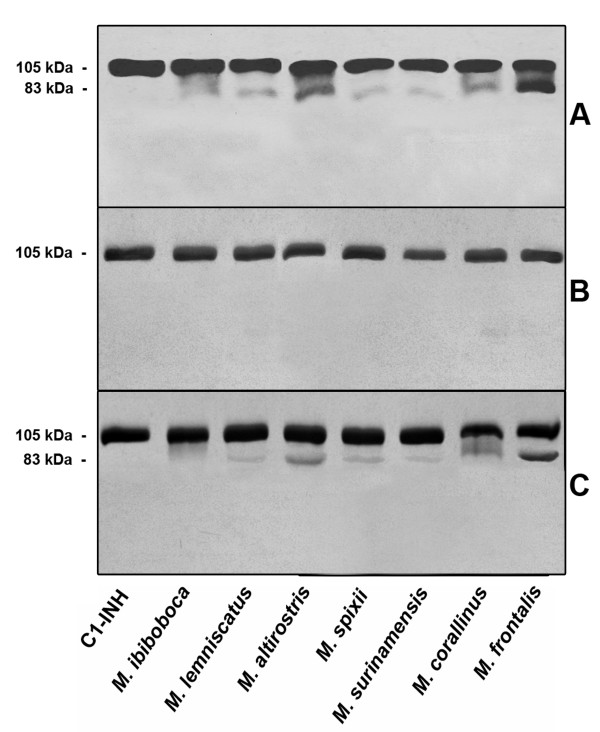
**Proteolytic action of the *Micrurus *spp venoms on human purified C1-INH**. **[A] **Samples of purified component C1-INH (1 μg) were incubated with 2 μg of *Micrurus *spp venoms or PBS for 30 min at 37°C. Inhibition assays were performed 10 mM of 1,10 phenanthroline **[B] **or PMSF **[C] **in the mixtures. Cleavage of C1-INH was visualized by western blot using anti-C1-INH serum.

Since the majority of *Micurus *venoms could interfere with the classical and/or lectin pathways, we have investigated if the complement regulator of activation of these pathways, C1-INH, would have undergone to cleavage. Figure 5A shows that all venoms induced cleavage of C1-INH, generating a main fragment of approximately 83 kDa. The fragmentation could be totally blocked by incubating venoms and C1-INH samples in the presence of 1,10 phenanthroline; PMSF could only partially abolish the cleavage of C1-INH induced by *M. ibiboboca *venom (Figure 5B and 5C).

## Discussion

The toxins present in venoms, used by snakes for effective immobilization of prey and for protection against predators, must rapidly reach the target organs. Therefore, mechanisms facilitating the distribution of the toxins throughout the tissues of the bitten animal must be activated. Hyaluronidase, which is present in almost all venoms, was the first of such mechanisms to be described [18]. Another mechanism which could fulfill this spreading function is local acute inflammation at the site of bite. Some potentially pro-inflammatory factors such as proteolytic enzymes and activators of clotting, complement and kallikrein-kinin systems have been detected in Crotalidae and Viperidae venoms. The action of these factors on the corresponding host substrates may release endogenous mediators of inflammation [14,19-22].

The Elapidae venoms analyzed in the present study from *Micrurus *genus were also able to activate the complement system. *M. ibiboboca *and *M. altirostris *venoms affected classical and lectin pathways; *M. spixii *and *M. frontalis *venoms affected only the classical pathway and *M. lemniscatus, M. surinamensis and M. corallinus *venoms only the lectin pathway. These differences of *Micrurus *spp venoms action on the complement system may be due to interspecies variations in venom composition. Indeed, previous results of our group [6] showed that venoms from these *Micrurus *species differ in protein composition and toxic potential, probably associated with the geographical origin, habitat and diet of the snakes.

The majority of the *Micrurus *spp venoms induced the production of significant amounts of the three anaphylatoxins C3a, C4a and C5a. This suggests that these venoms are able to activate the complement system and that the observed reduction of the CP and LP complement activities, after treatment with the various venoms, was most likely caused by activation of the pathways, resulting in consumption of the C-components; however some inactivation/inhibition of C-components may also have occurred.

The action of cobra venom factor (CVF) from the cobra *Naja naja*, as a complement activator and generator of anaphylatoxins, has been well described [14-16]. In the present study, the presence of CVF-like molecules was not detected in *Micrurus *spp venoms, using antibodies against *N. naja *CVF in western blot assays. These results may suggest that CVF components are not present in the *Micrurus *venoms here studied or that they do not share the same antigenic properties.

Analysis of a possible direct proteolytic action of *Micrurus *venoms on the complement molecules showed that, using purified C3, cleavage was induced by *M. ibiboboca, M. lemniscatus, M. corallinus, M. frontalis *but not by *M. altirostris, M. spixii *and *M. surinamensis*. C3 proteolytic activity in snake venoms from the Elapidae family has been previously reported by O'Keefe and collaborators [23] in *Naja naja siamensis *and by us in *N. naja, N. nigricollis *and *M. ibiboboca *[7], although no further characterization of the class of protease involved has been performed. In the present study, we have shown that metalloproteinases are the main proteases responsible for this event, since the specific inhibitor, 1,10 phenathroline, completely abrogated the C3α chain hydrolysis induced by *Micrurus *venoms, thus, possibly contributing to the activation and inactivation of the complement activation pathways, as well as to the generation of the anaphylatoxins. On the other hand, no cleavage of C4 was observed, suggesting the presence of a specific C3 α chain cleaving metalloprotease in some *Micrurus *venoms.

The majority of the *Micrurus *spp venoms induced consumption of the classical and or the lectin pathways and we investigated the mechanism of this. The presence of antibodies as initiating factor was discarded, since the human serum samples used were devoid of *Micrurus *spp venoms specific antibodies (data not shown). Another possibility investigated was the failure of the complement regulatory mechanisms. C1-INH is the primary regulator of the activation of the classical and lectin pathways and also plays an important role in the regulation of coagulation and fibrinolysis. C1-INH inactivates C1r and C1s, the serine protease subcomponents of the first component of the classical pathway, and MASP 1 and 2 proteases of the lectin pathway; removal/inactivation of C1-INH leads to auto-activation of the CP and LP. We show here that all *Micrurus *venoms were able to cleave the C1-INH, generating a fragment of approximately 83 kDa, and this hydrolysis could be blocked by metalloproteinase inhibitors 1,10 phenanthroline. PMSF could partially abolish the cleavage of C1-INH induced by *M. ibiboboca *venom.

Cleavage of C1-INH by Crotalid, Viperid and Colubrid snake venoms has previously been reported by Kress et al. [24], who demonstrated that the inhibitor was converted into an active intermediate species of 89-kDa and then a further cleavage resulted in formation of an 86-kDa inactive inhibitor. We have recently demonstrated that venoms snakes from *Bothrops *genus were also able to cleave C1-INH, generating a main fragment of approximately 83 kDa [17] and this fragmentation could be totally blocked by 1,10 phenanthroline. These data suggest that the different families of snakes may possess common factor(s) in their venoms allowing them to inactivate C1-INH, which may lead to unrestricted activation of both coagulation and complement cascades and participate in the mediation of the toxic effects of the venoms.

## Conclusion

Data presented here demonstrate that *Micrurus *venoms can activate the complement system, generating significant amounts of anaphylatoxins, which may assist, due to their vasodilatory effects, to enhance the spreading of other venom components.

## Methods

### Chemicals and reagents

Tween 20, bovine serum albumin (BSA), ortho-phenylenediamine (OPD), phenylmethylsulfonyl fluoride (PMSF), 1,10 phenanthroline, ethylene diamine tetracetic acid (EDTA), ethylene glycol bis-(β-aminoethyl ether)-N, N, N, N'-tetracetic acid (EGTA) and mannan were purchased from Sigma (St. Louis, Missouri, USA). Goat anti-rabbit (GAR) and rabbit anti-goat (RAG) IgG labelled with alkaline phosphatase (IgG-AP) or with horseradish peroxidase (IgG-HRPO), 5-bromo-4-chloro-3-indolyl-phosphate (BCIP) and nitroblue tetrazolium (NBT) were from Promega Corp. (Madison, Wisconsin, USA). Purified human C3 and C4, and goat IgG anti-human C4 were from Quidell Corporation (San Diego, CA, USA). Purified human C1-INH and rabbit IgG anti-C1-INH were from Calbiochem-Novabiochem Corp. (San Diego, CA, USA). Rabbit IgG anti-C3 was from Santa Cruz Biotechnology Inc. (Santa Cruz, CA, USA) and rabbit polyclonal serum against sheep erythrocytes was made in house. Polyclonal rabbit antiserum against *Naja naja *cobra venom factor (CVF) was a kind donation from Dr. Carl Wilhelm Vogel, Honolulu, Hawaii, USA.

### Venoms

Venoms from *Micrurus ibiboboca, M. lemniscatus, M. altirostris, M. spixii, M. surinamensis, M. corallinus, M. frontalis, Naja naja, Naja nigricollis, Naja mossambica and Naja melanoleuca *were supplied by Herpetology Laboratory from Butantan Institute, SP, Brazil. Stock solutions were prepared in PBS (10 mM sodium phosphate containing 150 mM NaCl, pH 7.2) at 1.0 mg/mL. The permission to access venoms from *Micrurus *spp snakes (permission no. 01/2009) was provided by the Brazilian Institute of Environment and Renewable Natural Resources (IBAMA).

### Human serum and erythrocytes

Human blood was obtained from healthy donors. Blood samples drawn to obtain sera were collected without anticoagulant and allowed to clot for 2 h at room temperature; the normal human serum (NHS) was stored at -80°C. Blood samples from sheep or rabbit, drawn to obtain erythrocytes for subsequent use as target cells, were collected in anticoagulant (Alsever's old solution: 114 mM citrate, 27 mM glucose, 72 mM NaCl, pH 6.1). The human blood samples in this study were obtained from three healthy donors in our research group who clearly knew the purpose of this study, according to the Blood Donation Law of Brazil. This study was approved by the ethics committee of Butantan Institute.

### Treatment of the normal human serum with *Micrurus *spp venoms

Samples of 50 μl of normal human serum (NHS) were incubated with 50 μl (50 μg) of *Micrurus *spp venoms or PBS. The mixtures were incubated for 30 min at 37°C and tested for the residual complement activity in hemolytic assays (for classical and alternative pathways measurements) or by ELISA (for lectin pathway evaluation).

### Haemolytic complement activity

For classical pathway analysis, sheep erythrocytes were washed in PBS and a 2% suspension was incubated with an equal volume of a 1:500 dilution in PBS of rabbit anti-sheep erythrocytes serum for 15 min at 37°C. The antibody-sensitized sheep erythrocytes were washed and resuspended in veronal buffered saline (VBS^++^: 3.7 mM Barbitone, 0.3 mM CaCl_2 _and 0.8 mM MgCl_2_, 145.5 mM NaCl, pH 7.2) at 2% final concentration. For alternative pathway analysis, rabbit erythrocytes were washed in PBS and resuspended in AP buffer (5 mM Na-barbital, 10 mM EGTA, 7 mM MgCl_2; _150 mM NaCl, pH 7.4) at 2% final concentration. The antibody-sensitized sheep erythrocytes or the rabbit erythrocytes (50 μl) were added to each well, of a 96-well plate, and incubated, for 30 min at 37°C, with venom- or PBS-treated NHS samples. For each sample to be tested doubling dilutions in VBS^++ ^were made (150 μl/well), including zero and 100% lysis wells. Unlysed cells were removed by centrifugation at 1300 rpm at 4°C for 5 min. Fifty microlitres of each supernatant were transferred to new 96-well plates containing 200 μl of water, and the absorbance measured at 414 nm as an index of hemolysis. Percentage of hemolysis for each well and the number of CH_50 _and AP_50 _for each serum were calculated by standard methods.

### Complement activation of the lectin pathway as determined by ELISA

Microtitre plates were coated with 10 μg/well of mannan, overnight at 4°C, washed three times with PBS/0.05% Tween 20, and blocked using 1% BSA in PBS for 30 min at 37°C. After washing, serial dilutions of NHS samples treated with PBS or *Micrurus *spp venoms were added. After 1 h of incubation at 37°C, plates were washed with BVB^++ ^(VBS^++^, pH 7.2, containing 0.1% BSA) and incubated with anti-human C4 (1:2.000) for 1 h at 37°C. Plates were washed three times with BVB^++^/Tween 0.05% and incubated with the specific anti-IgG antibody conjugated with HRPO for 1 h. Plates were washed and the reactions developed with OPD substrate according to the conditions established by the manufacturers (Sigma). The absorbances were recorded in an ELISA reader (Multiskan spectrophotometer EX, Labsystems, Finland) at λ492 nm. For complement activity ELISA, normal human serum arbitrarily set at 1000 aU/ml, was used to produce a calibration curve.

### Detection of Anaphylatoxins

Samples of normal human serum (50 μL) were incubated with PBS (50 μL) or with *Micrurus *spp venoms (50 μL = 50 μg) for 30 min at 37°C, and C3a/C3a desArg, C4a/C4a desArg, and C5a/C5a desArg concentrations were measured using the Cytometric Bead Array (BD Biosciences Pharmingen, New Jersey, California, USA). Anaphylatoxin capture beads were incubated with the standards or with the test samples, washed, and then incubated with phycoerythrin-conjugated detection antibodies to form sandwich complexes. Two-color flow cytometric analysis was performed using a FACSCalibur flow cytometer (Becton Dickinson Immunocytometry Systems, Santa Clara, California, USA). Data were acquired and analyzed using Becton Dickinson Cytometric Bead Array CBA software. Anaphylatoxin concentrations were determined from the standard curves, plotting anaphylatoxin calibrator concentration versus FL-2 mean fluorescence intensity.

### Proteolytic activity of *Micrurus *spp venoms on the complement molecules

Venoms samples (1 μg) were incubated with the human purified components C3, C4 or C1-INH (2 μg) in PBS for 30 min at 37°C, and the cleavage was detected by western blotting. 20 mM of PMSF or phenanthroline, inhibitors of serine- and metallo-proteases, respectively, were added to assess the nature of the cleaving enzyme in the venom.

### Electrophoresis and western blot

Samples were solubilised in reducing or non-reducing sample buffers and separated in 10% SDS-PAGE gels [25]. Gels were blotted onto nitrocellulose [26]. After transfer, the membranes were blocked with PBS containing 5% BSA for 2 h and incubated with anti-C3 (1:5000), anti-C4 (1:1000), anti-C1-INH human (1:2000) or anti-CVF (1:1500) sera, for 1 h at room temperature. Immunoreactive proteins were detected using GAR/IgG-AP or RAG/IgG-AP (1:7500) in PBS/1% BSA for 1 h at room temperature. After washing 3 times, for 10 min with PBS/0.05% Tween 20, blots were developed using NBT/BCIP according to the manufacturer's instructions (Promega).

## Abbreviations used

C: Complement system; CP: classical pathway; AP: alternative pathway; LP: Lectin pathway; C1-esterease Inhibitor: C1-INH; CVF: cobra venom factor; NHS: normal human serum.

## Authors' contributions

All authors have read and approved the final manuscript.

Conceived and designed the experiments: GDT, GPQ, MFDF, DVT. Performed the experiments: GDT, GPQ. Analyzed the data: GDT, GPQ, CWVB, DVT. Contributed with reagents/materials: MFDF, DVT. Wrote the paper: GDT, CWVB, DVT.
